# Supplementary material for: Non-Targeted Metabolic Profiling of Cerebellum in Spina Bifida Fetal Rats
Source: Metabolites. 2023 May 19;13(5):670. doi: 10.3390/metabo13050670 (PMC10223940; doi:10.3390/metabo13050670)
Supplement: Supplementary file 1 [file metabolites-13-00670-s001.zip › metabolites-2368211-supplementary.pdf]

Supplementary

Non-targeted metabolic profiling of cerebellum in spina bifida fetal rats

A.

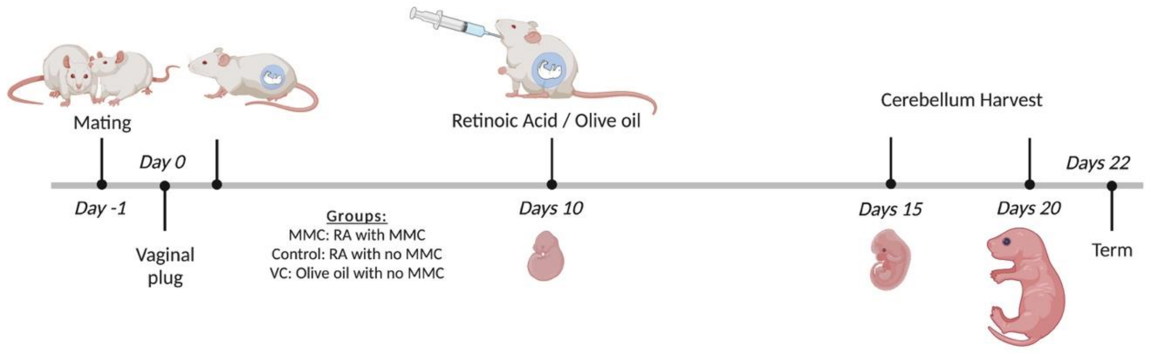

B.

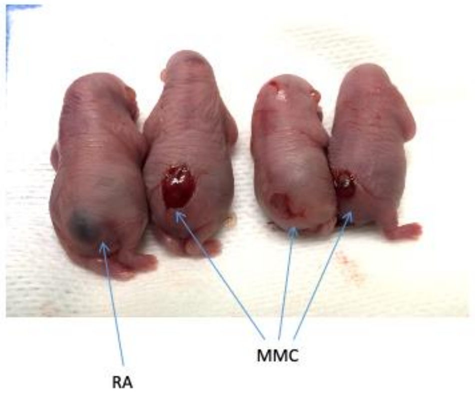

C.

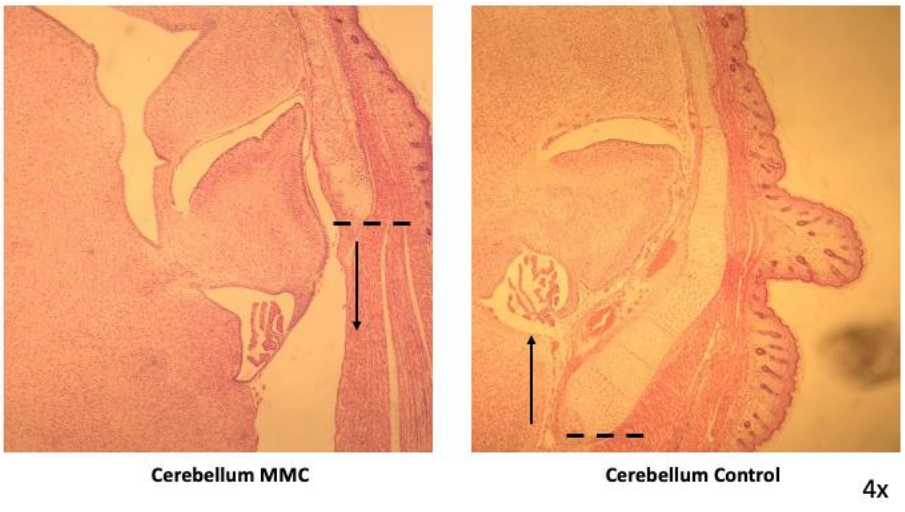

Supplementary Figure S1: A) Teratogenic congenital Retinoic Acid (RA)- Induced Spina Bifida rat model. B) Cerebellar tissue samples were collected and classified into one of three groups: spina bifida (labeled MMC), retinoic-acid sham (RA), or normal (VC) (not shown). C) Displacement of the cerebellum into de central canal in MMC rats compared to the Control. HE staining 4x

A.

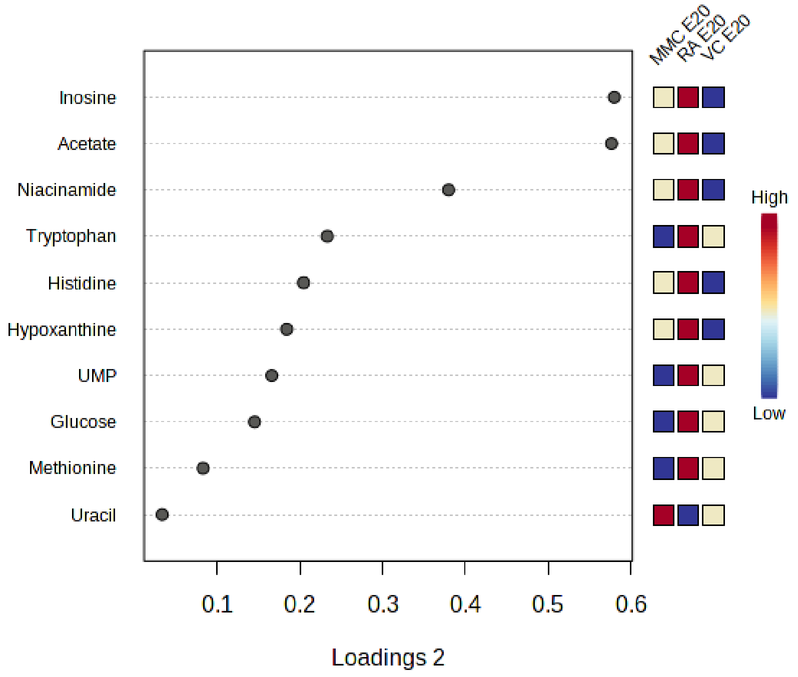

B.

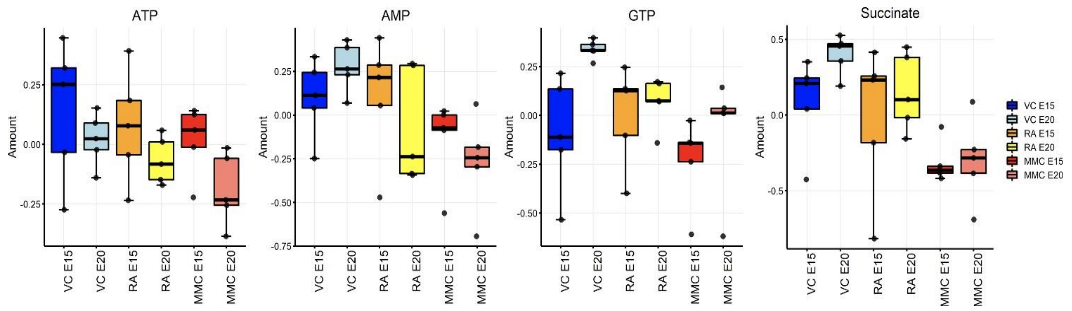

Supplementary Figure S2: A) Sparse partial least square discriminant analysis (sPLS-DA) loadings plot for Component 2 at late gestation (E20). B) Metabolites notable for RA contribution to variation between MMC and VC groups. Relative concentrations for four metabolites on sPLS-DA analysis at both mid (E15) and late (E20) gestation show potential for retinoic acid contribution to variations seen between VC and MMC groups.
